# quality of remission in the major depressive disorder

**DOI:** 10.1192/j.eurpsy.2023.1762

**Published:** 2023-07-19

**Authors:** H. Chebli, H. berrada, M. chtibi, S. belbachir, A. ouanass

**Affiliations:** 1 University Psychiatric Hospital Arrazi in Sale; 2 University Psychiatric Hospital Arrazi in Sale; 3 University Psychiatric Hospital Arrazi in Sale, SALE, Morocco

## Abstract

**Introduction:**

Depression is second only to cardiovascular disease as a cause of disability and affects 3 to 5% of the general population. The therapist always tries to guarantee a total remission of the symptoms of depression, but partial remission remains frequent.

**Objectives:**

Evaluating the quality of remission in a group of patients followed for major depressive disorder at the Arrazi Hospital in Salé.

**Methods:**

Cross-sectional study in adult patients followed up in consultation for major depressive disorder according to DSM-V criteria and having been under treatment for at least 2 months. Information was collected using a hetero questionnaire containing information on sociodemographic data and the depressive episode.

The quality of remission is assessed using the Hamilton Rating Scale for Depression.

**Results:**

We recruited 70 patients followed for major depressive disorder at 2 months of evolution. The Hamilton scale revealed that 40% of patients were in partial remission.

**Conclusions:**

Residual symptoms can be the cause of relapse in major depressive disorder, hence the interest in seeking both a symptomatic remission and a complete functional remission, and this by the evaluation and the continuous management of the patients.

**Disclosure of Interest:**

None Declared

